# Connectivity of larval stages of sedentary marine communities between hard substrates and offshore structures in the North Sea

**DOI:** 10.1038/s41598-018-32912-2

**Published:** 2018-10-03

**Authors:** Johan van der Molen, Luz María García-García, Paul Whomersley, Alexander Callaway, Paulette E. Posen, Kieran Hyder

**Affiliations:** 1 The Centre for Environment, Fisheries and Aquaculture Science (Cefas), Lowestoft, NR33 0HT UK; 2 Council of the Isles of Scilly, St Mary’s, Isles of Scilly, TR21 0LW UK; 3 The Centre for Environment, Fisheries and Aquaculture Science (Cefas), Weymouth, DT4 8UB UK; 4 NIOZ Royal Netherlands Institute for Sea Research, Dept. of Coastal Systems and Utrecht University, Den Burg, 1797 SZ The Netherlands; 5 School of Environmental Sciences, University of East Anglia, Norwich, NR4 7TJ UK

## Abstract

Man-made structures including rigs, pipelines, cables, renewable energy devices, and ship wrecks, offer hard substrate in the largely soft-sediment environment of the North Sea. These structures become colonised by sedentary organisms and non-migratory reef fish, and form local ecosystems that attract larger predators including seals, birds, and fish. It is possible that these structures form a system of interconnected reef environments through the planktonic dispersal of the pelagic stages of organisms by ocean currents. Changes to the overall arrangement of hard substrate areas through removal or addition of individual man-made structures will affect the interconnectivity and could impact on the ecosystem. Here, we assessed the connectivity of sectors with oil and gas structures, wind farms, wrecks, and natural hard substrate, using a model that simulates the drift of planktonic stages of seven organisms with sedentary adult stages associated with hard substrate, applied to the period 2001–2010. Connectivity was assessed using a classification system designed to address the function of sectors in the network. Results showed a relatively stable overall spatial distribution of sector function but with distinct variations between species and years. The results are discussed in the context of decommissioning of oil and gas infrastructure in the North Sea.

## Introduction

There are many potential influences of man-made structures on marine ecosystem structure and function, and these include impact on habitat connectivity (e.g. stepping stones, pelagic dispersal, linkage^1^) and movement of mobile marine species (e.g. crabs^2^, fish^3^, birds^4^, seals^5^). Man-made structures may also support communities that are different to those found on natural substrate, so affecting the function of the ecosystem^6^. In fact, addition to the marine environment of man-made structures like oil platforms, wind turbines, and shipwrecks can be positive (e.g. strengthening natural connections between habitats and MPAs), or detrimental by introducing conduits for non-native species^7^.

The connectivity of many marine systems has been assessed including coral reefs^8^, intertidal rocky shores^9,10^ and fish^11^. Many marine systems have been described as ‘open’^12,13^ and have been shown to have the potential for dispersal over large areas and protracted time frames during the pelagic phases of many of the marine organisms^14^. More recently, the impact of pelagic dispersal on connectivity has been studied using particle tracking approaches that model both hydrodynamics and larval behaviour. These models have been used to assess recruitment of commercial fish species^15–20^ and jellyfish^21^. Corridors between structures such as pipelines provide a mechanism for colonisation of reef species that do not have pelagic dispersal^6^. The behaviour of mobile organisms is also important and has been extensively studied^5,11^.

Decommissioning of man-made structures at the end of their use is generally a condition of the licence to operate^22–25^. In the North Sea, oil and gas platforms are coming to the end of their life and the estimated cost of decommissioning oil and gas infrastructure between 2016 and 2025 is £17.6 billion^26^, with the best estimate of cost for the UK continental shelf to 2050 of £47 billion^27^. There are many different options for decommissioning structures, ranging from complete removal to leaving in place or dumping at sea. There are legal frameworks specifying the level of removal required^22^, but derogations may be granted on health and safety, economic, social or environmental grounds^23^. For example, in the Northeast Atlantic, the decommissioning of offshore installations is regulated under OSPAR 98/3. This states that dumping or the leaving of disused offshore installations in place is prohibited, but derogations may be granted where an alternative disposal method is preferable to reuse or recycling or final disposal^23^. Derogations can include footings of a steel installation, concrete installations or anchors, or circumstances resulting from structural damage or deterioration^23^. In practice, this is likely to mean that all topsides and substructures of less than 10,000 tonnes will be removed and brought to shore for recycling, with derogations for structures of greater than 10,000 tonnes for footing, heavy concrete gravity-based structures, and any concrete anchor-base, which can then be left in place.

Derogations may be granted if there are thought to be benefits for the marine environment (e.g. Rigs-to-Reefs), but there is debate about whether the effect is beneficial for the marine environment^1,28,29^. In the North Sea, the majority of the seabed in made up by mud and sand, with rocky shores in many places and some reefs. Offshore installations and other man-made structures (e.g. wrecks) may provide hard substrate that impacts on the ecosystem in terms of productivity of the system and the interconnectedness of the network of hard substrates. Hence, it is important to understand the potential effects of man-made structures on the ecosystem and take these into account when deciding on appropriate decommissioning strategies. In the North Sea, this involves connectivity (pelagic dispersal, linkage by pipelines) and the movement of mobile predators. However, studies of the contribution of oil and gas infrastructure to the network of natural hard substrate are lacking.

This paper focusses on connectivity through planktonic life stages of several different sedentary species commonly found in association with man-made structures using an existing particle tracking model^20,21^. The General Individuals Transport Model (GITM) includes physical particle advection based on current fields from hydrodynamic models, diffusion, biological development, and behaviour^18,21^. The biological development and behaviour module includes egg and larval development, and vertical migration behaviour. The particle tracking runs were set up in a generic and schematised way to facilitate maximum comparability between the results for different species, and maximum flexibility (various forms of scaling, decommissioning scenarios) for a subsequent in-depth network analysis. As a result of these simplifications and generalisations, the results presented here, while capturing the main features of connectivity, may not necessarily have the highest potential level of detail. The results are discussed in the context of decommissioning strategies for oil and gas infrastructure in the North Sea.

## Results

### Example of post-processing

**Figure 1:**
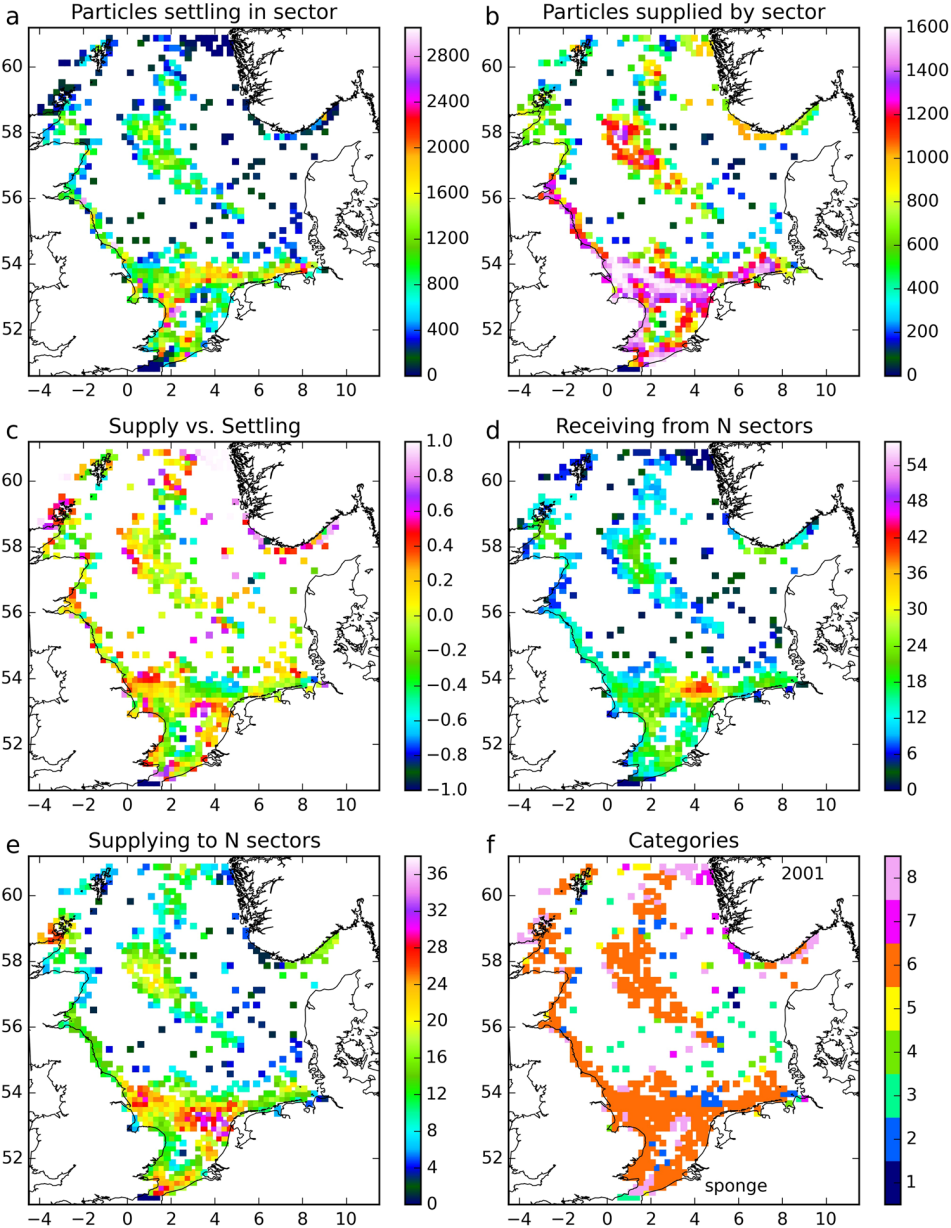
Connectivity results for sponges for 2001. (**a**) Number of particles potentially settling in each sector; (**b**) associated number of particles supplied by each sector; (**c**) mixture of supply and settling roles per sector; (**d**) number of sectors from which a sector received; (**e**) number of sectors to which a sector supplied; (**f**) connectivity category of each sector: 1: mono-receivers; 2: multi-receivers; 3: combined mono-suppliers, mono-receivers; 4: combined mono-suppliers, multi-receivers; 5: combined multi-suppliers, mono-receivers; 6: combined multi-suppliers, multi-receivers; 7: mono-suppliers; 8: multi-suppliers. The figure was created using Python 2.7 (www.anaconda.com/download).

To illustrate the post-processing procedure leading to classification into connectivity categories, we consider the maps for sponges in 2001 (Fig. 1). Each map is the result of one of the steps introduced in Methods. Sponges were selected because the results showed more diversity in resulting categories than other species. For this illustration we consider a single year.

Step 1 counted the number of particles potentially settling in each sector (Fig. 1a). Relatively large numbers of particles were ready to settle when passing through sectors in shallow areas of the southern North Sea. Moderate numbers of particles were in settling stage in sectors containing oil and gas structures in the central to northern North Sea and in sectors along the northeast coast of the UK. Low numbers were recorded in the areas between these, i.e., the offshore parts of the German Bight; the northeastern area towards Scandinavia; and in the vicinity of the open boundaries of the domain used for the particle tracking runs in the far north and the Straits of Dover.

Step 2 recorded the origin of these particles that were ready to settle and the number of particles supplied to the network by each sector (Fig. 1b). High numbers occurred in a band along the east coast of the UK and crossing over and along the continental coasts in the southern North Sea. High numbers were also recorded in the sectors containing oil and gas structures in the central to northern North Sea. Moderate numbers occurred along the Norwegian coast, and low numbers were simulated for particles originating in the far north and in a band surrounding the central sectors. As sponge larvae have a relatively short pelagic phase (20 days, Table 1), this relative similarity in spatial patterns of origin and settling is to be expected.

**Table 1:** Species life-history parameters in the model^31,80^.

Species	Total duration^30,31,80,82–84^	Spawning^31,80,84^	Egg stage^32,82,83,85^		Larval stage 1^32–34,86–88^				Larval stage 2^33,34,85–87,89–91^		
	[days]	Peak date and standard deviation	stage duration [days]	vertical migration	initial size [mm]	growth rate [mm/day]	vertical migration	final size [mm]	growth rate [mm/day]	vertical migration	final size [mm]
Dead man’s fingers (*Alcyonium digitatum*)	200	1 Jan 22 days	7	neutral	0.6	0.0015	neutral	0.615	0.0015	sinking 5 mm/s	0.9
Edible sea urchin (*Echinus esculentus*)	50	15 Apr 35 days	1.5	neutral	0.6	0.0071	neutral	0.72	0.0071	sinking 5 mm/s	0.88
Cold water coral (*Lophelia pertusa*)	57	15 Feb 56 days	7	floating 5 mm/s	0.5	0.0714	floating 5 mm/s	1.5	0.0714	sinking 5 mm/s	4.07
Plumose anemone (*Metridium senile var. dianthus*)	180	15 June 15 days	0.1	neutral	0.5	0.0055	neutral	0.722	0.0055	sinking 5 mm/s	1.5
Sponges (Porifera)	20	15 May 15 days	5	neutral	0.2	0.015	floating 5 mm/s	0.275	0.015	sinking 5 mm/s	0.5
Blue mussels (*Mytilus edulis*)	60	1 Aug 30 days	5	neutral	0.6	0.0053	floating 5 mm/s	0.75	0.0053	tidal, up flood, down ebb, 5 mm/s	0.9
Slipper limpet (*Crepidula fornicata*)	21	1 Jun 90 days	5	neutral	0.5	0.0714	sinking 5 mm/s	—	—	—	2.0

Growth rates were constant and based on larval stage duration.

Step 3 calculated the settling/supply factor *R* for each sector (Fig. 1c). Most sectors recorded moderate values, suggesting a mixed role in the network. A limited number of sectors were dominant suppliers, and were located near the Norwegian coast, in the far north and around the Orkney Islands. There were also some scattered sectors in the central North Sea and in the southern Bight. Very few sectors recorded very low values associated with dominant settling.

Step 4 counted the number of sectors from which a particular sector received particles (Fig. 1d). Most sectors received particles from between 10 and 30 other sectors. A limited region off the northwest coast of The Netherlands received from 30–40 sectors, and a band surrounding the central sectors containing oil and gas structures, including the far north, received from very few sectors.

Step 5 derived, in a similar way, the number of sectors to which a sector could supply particles (Fig. 1e). A band of sectors across the southern North Sea supplied to more than 25 other sectors, while the central sectors with oil and gas structures supplied to 10–20 other sectors. Sectors in the band surrounding these central sectors typically supplied to very few other sectors.

Step 6, finally, classified the sectors in connectivity categories (Fig. 1f). Most sectors were found to be multi-suppliers and multi-receivers (Category 6). The northeast of the domain contained a substantial number of sectors classed as dominant suppliers (a mix of Categories 7, mono-suppliers, and 8, multi-suppliers). The band surrounding the central region with oil and gas structures contained a substantial number of sectors in Category 3 (mono-suppliers and mono-receivers). Within this main pattern, there was a scatter of individual or small clutches of sectors of other categories.

### Results per species

**Figure 2:**
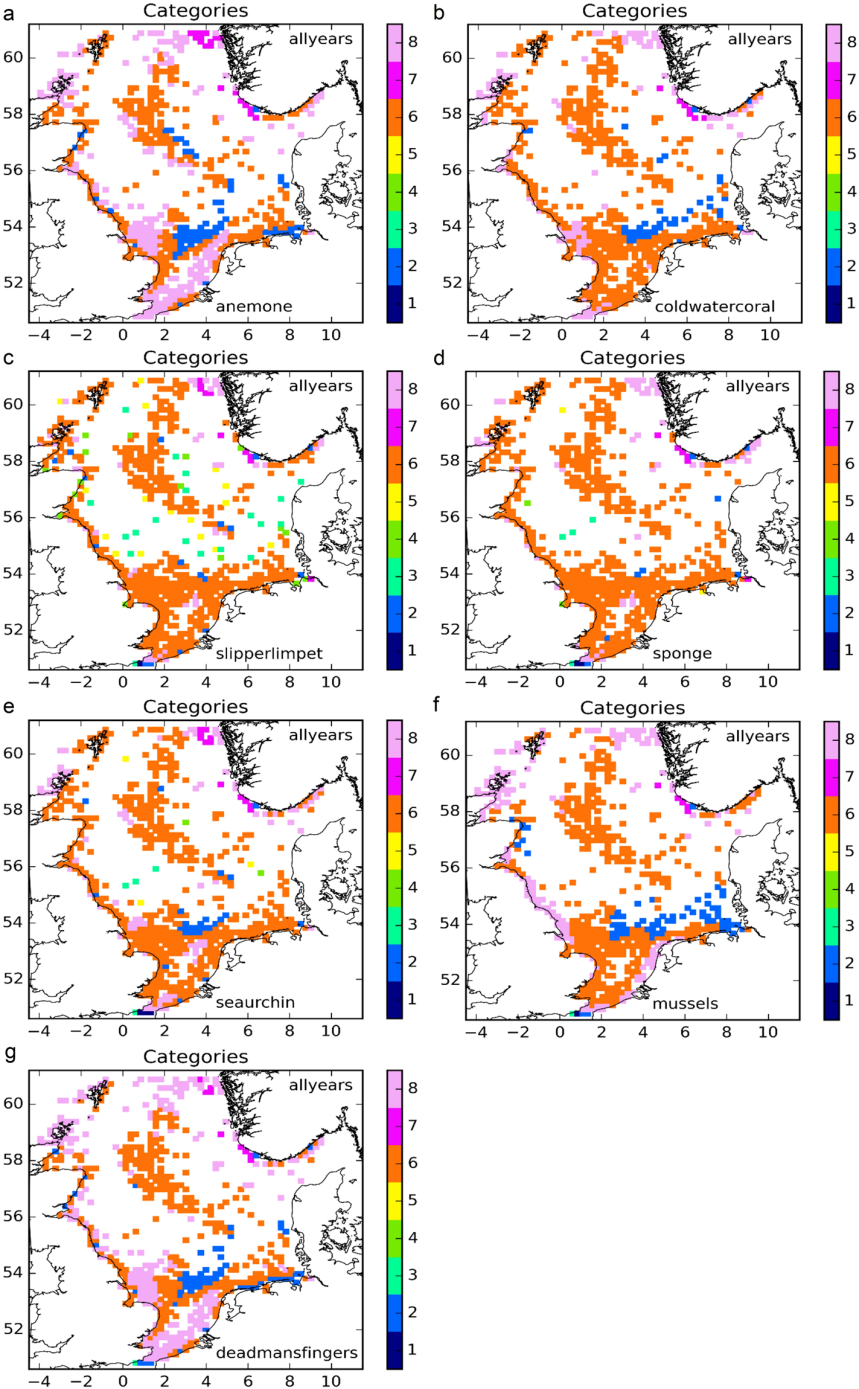
Functional categories, aggregated over 2001–2010 for (**a**) anemone, (**b**) cold water coral, (**c**) slipper limpet, (**d**) sponges, (**e**) sea urchin, (**f**) mussels, (**g**) dead man’s fingers. The figure was created using Python 2.7 (www.anaconda.com/download).

For each species, results were aggregated over the 10 years of the simulations, and post-processed into connectivity categories (Fig. 2). A full set of plots, as in Fig. 1, is included in the supplementary material.

For plumose anemone (*Metridium senile*) (Fig. 2a) Sectors of Category 8 (multi-suppliers) were found along the east coast of the UK and in the southern Bight, and along the continental coast, along the Norwegian coast, to the west of the Orkney Islands, on the western side of the central region with oil and gas structures, in the vicinity of the Silver Pit, and in the northern approaches to the Strait of Dover. The coastal region of Norway also contained sectors of Category 7 (mono-suppliers). Three bands of Category 2 (multi-receivers) were found: in the frontal area in the southern North Sea, to the east of the central region with oil and gas structures, and along the German coast.

Cold-water coral (*Lophelia pertusa*) showed a comparable pattern, but with reduced presence of sectors in Category 8 (multi-suppliers), and only a single patch of Category 2 (multi-receivers) in the frontal area in the southern North Sea, which extended almost to the Danish coast (Fig. 2b). While it is clear that *Lophelia pertusa* needs deeper water than found in the southern North Sea, the connectivity identified for the southern North Sea relates to any species with similar larval characteristics.

Slipper limpet (*Crepidula fornicata*) (Fig. 2c) showed dominance of Category 6 (multi-suppliers and multi-receivers) sectors. Exceptions were a region of sectors of Categories 7 (mono-suppliers) and 8 (multi-suppliers) along the Norwegian coast, and a band surrounding the central region with oil and gas structures, in which a substantial number of sectors fell within Categories 3 (mono-suppliers, mono-receivers) and 5 (multi-suppliers, mono-receivers).

Results for sponges (Porifera spp.) (Fig. 2d) were similar to those of slipper limpet, except that there was not much evidence of a central band representing Categories 3 and 5.

Edible sea urchin (*Echinus esculentus*) (Fig. 2e) displayed a pattern that could be considered a cross-over between slipper limpet and cold-water coral. There was a reduced region with sectors of Category 2 (multi-receivers) near the frontal region, and weak evidence of a band with Categories 3 (mono-suppliers, mono-receivers) and 5 (multi-suppliers, mono-receivers) surrounding the central region with oil and gas structures.

Blue mussels (*Mytilus edulis*) (Fig. 2f) had a substantial number of sectors of Category 8 (multi-suppliers), located around the Orkney and Shetland Islands, the east coast of the UK, the west coasts of Belgium, the Netherlands and Norway. An extensive band of sectors of Category 2 (multi-receivers) stretched along the frontal area and into the German Bight, all the way to the coast.

The results for dead man’s fingers (*Alcyonium digitatum*) (Fig. 2g) were similar to those of mussels, but with increased occurrence of sectors of Category 8 (multi-suppliers) stretching further off-shore, and a smaller region of sectors of Category 2 (multi-receivers) along the frontal area. There was an additional strip of sectors of Category 2 (multi-receivers) along the northern coasts of The Netherlands and Germany. Overall, the seven species displayed similarities in the overall pattern of categories of connectivity, but with substantial individual differences.

### Results for the community

**Figure 3:**
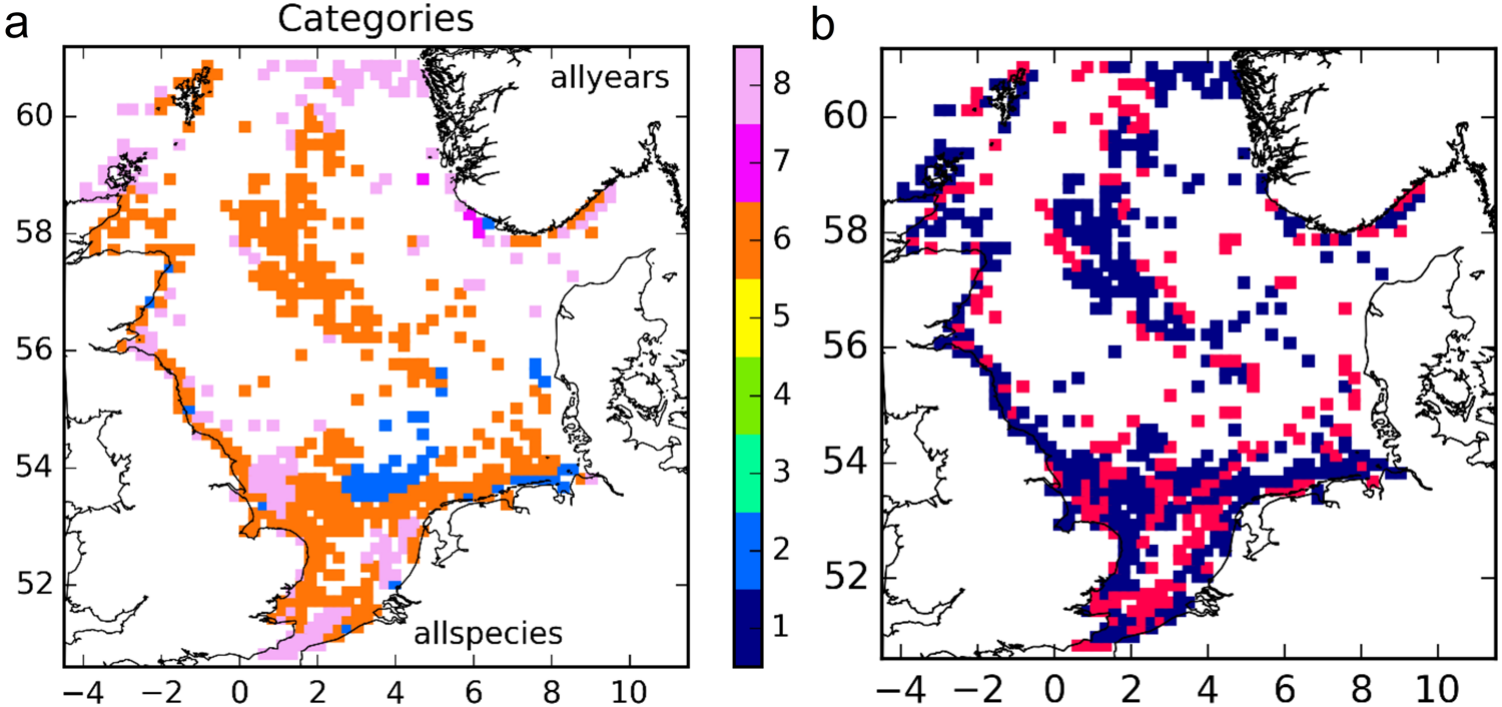
Community results. (**a**) Functional categories, aggregated over all species and all years; (**b**) sectors with the same category in all years (blue), and that changed category at least once (red). The figure was created using Python 2.7 (www.anaconda.com/download).

Offshore structures contain a community of species, all of which contribute to the network, and all of which will be affected if structures are added or removed. To obtain, as far as the model results allow, some insight into this community network, the results were aggregated for all modelled species for all the years (Fig. 3a). Patches of multi-suppliers (Category 8) were found predominantly in areas relatively close to coasts, but also offshore in the far North. Multi-suppliers, multi-receivers (Category 6) were found elsewhere, except for an area of multi-receivers (Category 2) in the central southern North Sea and near the German coast. Many sectors changed category between years at least once according to meteorological differences, however, in particular near boundaries between patches of categories (Fig. 3b).

## Discussion

We ran a series of particle tracking model experiments and analysed them in terms of connectivity between sectors with structures and hard substrate in the North Sea, with the aim to contribute useful information for decisions about decommissioning of oil and gas structures. In the following, we first discuss the individual species. Then, as oil and gas structures host many species and decommissioning affects them all, we discuss the modelled species aggregated together. Finally, we suggest how this information could support decommissioning decisions.

Not all the species used here occupy or can survive in the full geographic range used in this model study. We have nevertheless assumed that they do, and ignored survivability, because other, related species with similar life-history characteristics may have different spatial distributions, and the aim was to produce a fully inter-comparable set of simulations. The differences in connectivity category distributions between the species were caused by their different characteristics (in particular spawning time, pelagic duration, and vertical migration behaviour; Table 1), and how these interacted with the time- and space-varying currents. The intricacies of these interactions cannot be presented here, but a few observations can be made.

For instance, dead man’s fingers (*Alcyonium digitatum*) showed a predominance of Category 8 (multi-suppliers) in the southern North Sea. This is understandable, considering that this species had the longest pelagic duration (180 days), and spawned in winter^30^ when winds are strong, typically from westerly directions, setting up a counter-clockwise circulation. Hence, there is a high chance for particles released in these areas to be transported out of this area.

Another example is the absence of the region of sectors of Category 2 (multi-receivers) along the frontal area in the southern North Sea for slipper limpet (*Crepidula fornicata*) and sponges (Porifera spp.). These two species have the shortest pelagic duration (about 20 days) of all species considered here, and also spawn in early summer when meteorological conditions are relatively quiet^31,32^. As a result, particles released from that area have a chance to be retained, resulting in local supply and settling, whereas other species are more likely to be transported away towards the northeast. In that direction, there are few sectors with structures on which to settle, reducing the opportunity for their parent sectors to act as suppliers to the network for the other species.

The short pelagic duration is also most likely responsible for the Category 3 (mono-suppliers, mono-receivers) sectors in the band around the central region for slipper limpet (*Crepidula fornicata*) (and to a lesser extent for sponges; Porifera spp.): these sectors, being relatively isolated, derive particles from a few nearby upstream sectors, or even from themselves.

Mussels were the only species with many sectors of Category 2 (multi-receivers) in the inner German Bight. The particles representing mussel larvae had tidally-induced vertical migration behaviour in the last larval stage, which is a very effective mechanism to transport larvae in the direction of flood tidal currents^33,34^. In the German Bight, tidal currents have a dominant axis directed into the Bight^35^. This dominant axis of flood and ebb flow, in combination with the tidally cued vertical migration behaviour of the mussel larvae (up during flood flow and down during ebb flow), resulted in larger numbers of particles settling in that area than for the other species.

Simply adding the standardised results for the selected species as modelled to obtain an indication of community connectivity is a coarse approximation. In reality, these results can be expected to be modulated by differences in fecundity, survival (potentially related to larval duration), geographic range, available habitat per sector, contributions by distant populations outside the model domain, available space for colonisation in the habitat^12,13,36^, movement of adults (e.g. along pipe lines^6^), and the presence of species in addition to those included here. These caveats could not be addressed within the current simulations because of lack of information and resource limitations. Nevertheless, some observations can be made. Comparing Fig. 3 with Fig. 2 suggests that recognisable elements of the patterns of all species are present in the composite. It also suggests that, as may be expected when adding heterogeneity, only categories with ‘multi’ labels are retained, essentially simplifying the categories back into the main classes of suppliers, conductors and receivers. If further detail were added (more species, more years, and more variety), these trends are likely to be consolidated.

We now discuss the importance of the various categories of sectors for the functioning of the network. Receivers are the end-members of the network, and removal would not result in changes to the wider network. Suppliers may be key ‘anchor points’ for the network, and removal may reduce populations in other sectors of the network. They may also be more vulnerable to disturbance than Receivers and Conductors, as they are likely to rely on self-seeding to maintain the population, and are hence less likely to recover. Multi-suppliers supply larvae to a larger part of the network than Mono-suppliers. Mono-receivers are more vulnerable than Multi-receivers as they have fewer sources that can provide larvae for recolonisation after disturbance. Note, however, that based on the discussion above, mono-types are likely to be very rare at a community level. This interpretation suggests that the network is probably most sensitive to removal of structures in sectors of Categories 7 and 8 that act as anchor points in the model. Removal of substantial numbers of structures of Category 6 may result in fragmentation of the network.

**Figure 4:**
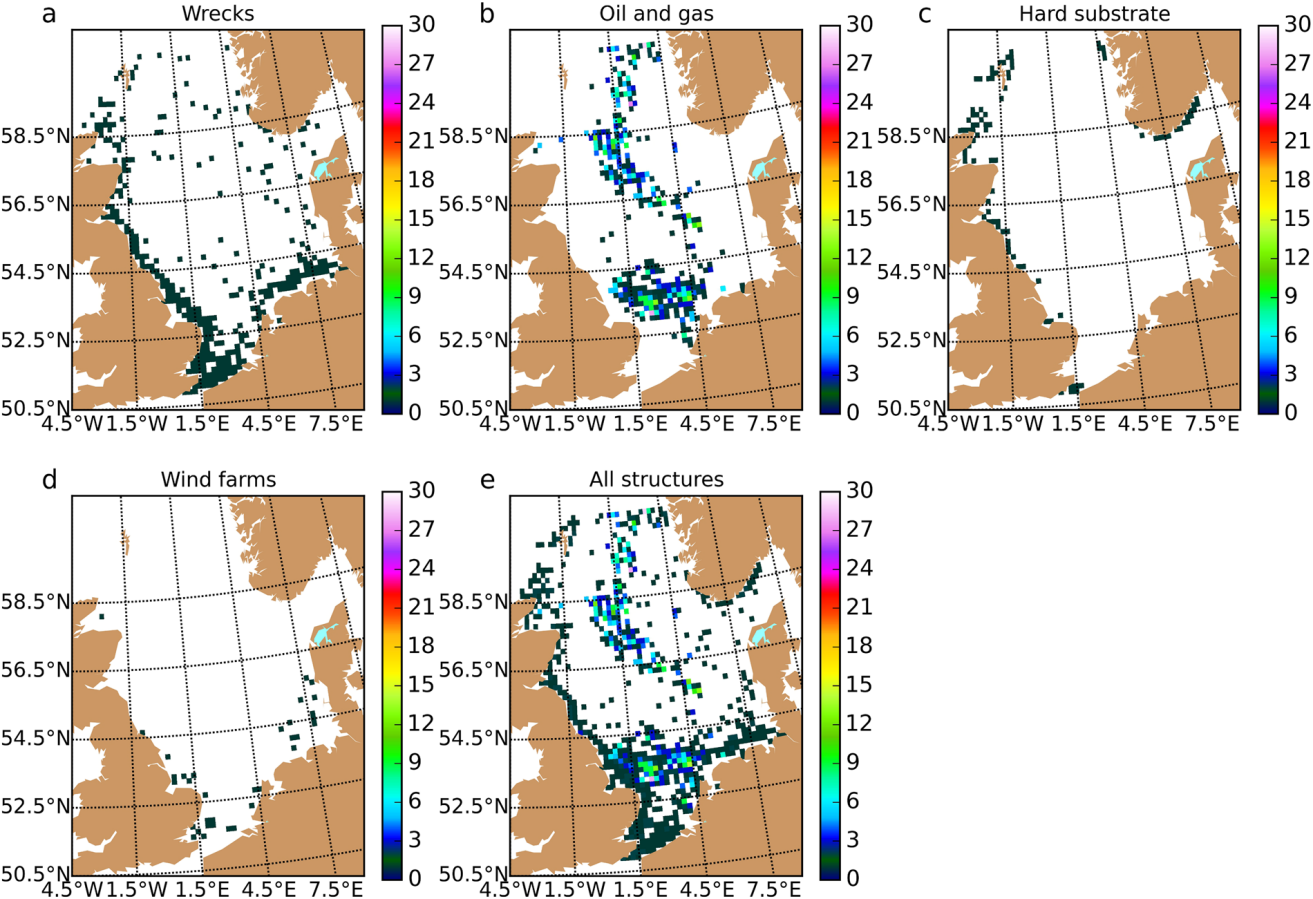
Sectors with (**a**) wreck tonnage above the threshold, (**b**) oil and gas structures, (**c**) natural hard substrate, (**d**) wind farms, and (**e**) combined. Colours indicate the number of structures. Wrecks, wind farms and hard substrate were counted as one structure per sector. The figure was created using Python 2.7 (www.anaconda.com/download).

Comparison of the distribution of oil and gas structures (Fig. 4b) with the decadal-aggregated categories of the various species (Fig. 2) suggests a number of regions where removal or retention may have the largest effect: (a) in the far northeast (60–61N, 2–5E), (b) along the western edge of the central region (57–59N, 1–0W), (c) on the Norfolk Banks (53–54.5N, 1–2E), and (d) off the north-west coast of The Netherlands (52–53.5N, 4–5E). The region in the far northeast (a) is near the boundary of the model region used for particle tracking, hence results for that area are likely to be less reliable and not further discussed here. The region with instances of sectors of Category 8 along the western edge of the central region (b) may act as an anchor point for several of the species considered here. The areas Norfolk Banks (c) and off the northwest coast of The Netherlands (d) also contain wrecks (Fig. 4a), hence may be less sensitive to removal of oil and gas structures. For Conductors, an investigation of the level at which thinning of oil and gas structures will start to affect the functioning of the network through the creation of isolated communities would be sensible further work. Finally, it is conceivable that some of the more isolated sectors with oil and gas structures provide links within the network that can be severed very easily; these also deserve further attention.

The model results have provided a first assessment of larval connectivity between sectors with natural hard substrate and offshore structures, and suggested several regions in the current network in which removal or retention of structures may give a different network response in terms of this connectivity. This study provides a contribution to the ensemble of biological, ecological, environmental, technical, economic, societal, legal and political information and evidence needed to make balanced decisions on decommissioning of offshore structures, either in general terms or for specific cases, and can inform the discussion. The direction of this discussion (preservation of network features to, e.g., support existing biological communities, or disruption of network function to, e.g., reduce the potential spreading of non-native species^7^) is not the subject of this paper.

## Methods

### Study area

**Figure 5:**
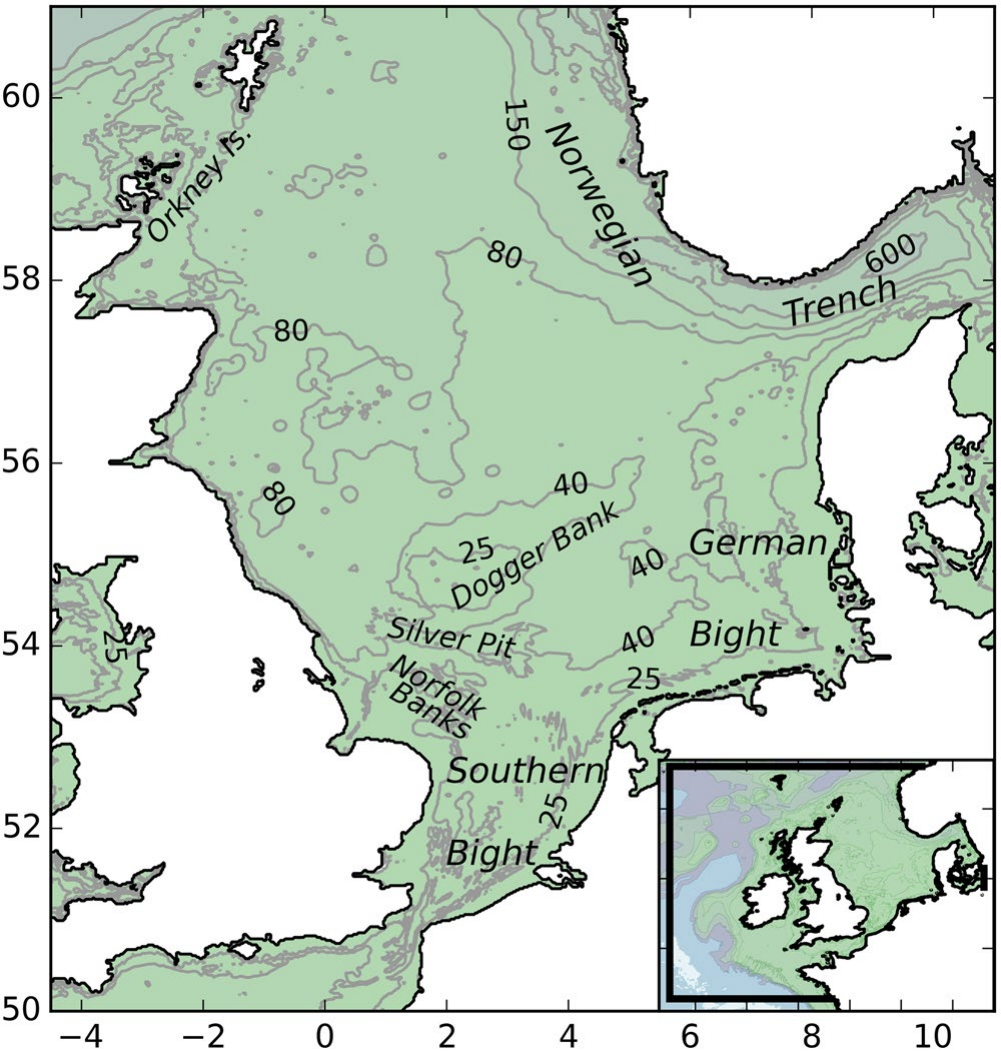
Study area with bathymetry, depth in m. Inset: spatial domain of the hydrodynamics model. The figure was created using Python 2.7 (www.anaconda.com/download), earlier versions were used by Van der Molen and colleagues^49^ © Authors 2016, and Van der Molen and colleagues^50^ © Crown 2016.

The North Sea (Fig. 5) is a shallow shelf sea with water depths that range from 20 to 600 m, but averaging approximately 70 m in the south increasing to 100 m in the central and northern regions. The glacial ice-pushed ridge that forms Dogger Bank separates the central and southern parts of the North Sea. The Norwegian Trench and Skagerrak separate these areas from the Scandinavian coast with depths of several hundreds of metres. An overview of the hydrography of the North Sea was compiled by Otto and colleagues^37^. The tides are predominantly semi-diurnal, with ranges of up to several metres along the coasts, and M_2_ amphidromic points in the Southern Bight, the German Bight and near the southern tip of Norway^38,39^. Model-based estimates were derived by Holt and colleagues^35^, and also by Davies and colleagues^40^, including other tidal components. Tidal currents have been shown to reach speeds of over 1 ms^−1^ in coastal areas^35,41^. The central and northern North Sea stratify in summer, while the southern area remains well-mixed^42,43^. The residual circulation is generally clock-wise, with inflows along the Atlantic boundary in the North and through the Strait of Dover^44^, but with seasonal variations^35^. Contributions by wind and tides are of comparable magnitude^39^. During stratified conditions in summer, subsurface jet-like currents occur near the thermocline around the Dogger Bank^45,46^. Tracing of accidental radioactive releases in the 1970 s indicated that it can take several years for waters to pass through the North Sea^47^.

### GETM: North-west European Shelf setup

The 3D hydrodynamic model GETM (General Estuarine Transport Model^48^, www.getm.eu) solves the shallow-water, heat balance, and density equations. It uses GOTM (General Ocean Turbulence Model) to solve the vertical dimension. GETM was run using the north-west European shelf setup used to study the potential largescale effects of tidal energy generation in the Pentland Firth^49^ and to develop a suspended particulate matter model^50^. The setup includes a spherical grid covering the area 46.4°N–63°N, 17.25°W–13°E with a resolution of 0.08° in longitude and 0.05° in latitude (approximately 5.5 km), and 25 non-equidistant layers in the vertical. The model bathymetry was based on the NOOS bathymetry (www.noos.cc/index.php?id=173). The model was forced with tidal constituents derived from TOPEX-POSEIDON satellite altimetry^51^, atmospheric forcing from ECMWF ERA-Interim^52,53^ (www.ecmwf.int/en/research/climate-reanalysis/era-interim), interpolated river runoff from a range of observational data sets (the National River Flow Archive (www.ceh.ac.uk/data/nrfa/index.html) for UK rivers, the Agence de l’eau Loire-Bretagne, Agence de l’eau Seine-Normandie and IFREMER for French rivers, the DONAR database for Netherlands rivers, ARGE Elbe, the Niedersächsisches Landesamt für Ökologie and the Bundesanstalt für Gewässerkunde for German rivers, and the Institute for Marine Research, Bergen, for Norwegian rivers; see also Lenhart and colleagues^54^), and depth-resolved temperature- and salinity boundary conditions from ECMWF-ORAS4^55,56^ (http://www.ecmwf.int/products/forecasts/d/charts/oras4/reanalysis/).

### General Individuals Transport Model

The Individual Behaviour Model (IBM) GITM includes physical particle advection and diffusion, and biological development and behaviour. The advection-diffusion elements of GITM were based on a re-coded version of the Lagrangian semi-analytical advection-diffusion method that ensures particles follow stream lines^57^. Furthermore, a random walk method with advective correction^58^ was included to simulate diffusion^59^. This method uses a constant diffusion coefficient in the horizontal direction and a variable diffusion coefficient in the vertical direction. The latter is based on the vertical diffusivity obtained from the turbulence closure model in the hydrodynamics model GETM. The combined hydrodynamics model (GETM) and particle tracking model (GITM) were applied recently to simulate the transport of plaice larvae^20^ and population dynamics of *Mnemiopsis leidyi*^21^ in the North Sea, the latter also using food fields from the biogeochemical model ERSEM^60–68^ (www.nioz.nl/en/about/cos/coastal-systems-modelling). The biological development and behaviour module of GITM allows particles to progress through a user-defined number of egg and larval development stages. Egg stages are defined by temperature-normalised egg duration, and larval stages are defined by size. Stage progression depends on daily development (eggs) and growth (larvae) rates, which can be prescribed, for each stage, as constants or derived from temperature-dependent formulations. During each development stage, a particle can have stage-specific vertical migration behaviour (neutrally buoyant, floating, sinking, diel migration, tidally cued migration). Particles can be considered as super individuals, representing a large number of individuals that can be subject to mortality functions (not used here). GITM runs off-line, using stored hydrodynamics from GETM at hourly intervals to account for the effect of tides. A set of automated scripts was used to split an individual model run into multiple jobs, process them in parallel on a linux cluster and combine the results at completion. Multi-threading was also introduced to the most computationally intensive loops to speed up the computations.

### Offshore structures and natural substrates

A dataset of natural substrates and man-made structures was compiled to support the modelling processes. Different types of substrate and structure have the capacity to support a variety of marine communities, but environmental requirements will differ between species. In many cases, it is not merely the presence of a certain type of substrate or structure that will determine the establishment and continued success (or otherwise) of a community, but also the spatial extent and/or connectivity of those features. In order to assess the extent to which man-made structures increase connectivity between naturally-occurring hard substrate features in the North Sea, and what influence their presence has on marine communities, it was necessary to examine the spatial relationships between these features. Spatial data were processed and analysed using ArcMap v10.1 (http://www.esri.com/).

Natural substrate data were compiled primarily from the EMODnet Phase II Seabed Habitats (October 2015) 1:250,000 vector layer (www.emodnet.eu/seabed-habitats) using the FOLK_5cl habitat classes^69,70^, representing the most up-to-date high-resolution natural substrate data available to us at the time of the study. This layer contained ‘No Data’ areas that were under revision subject to ongoing survey and modelling work. Therefore, it was necessary to fill these data gaps with, firstly, the lower resolution EMODnet Phase II Seabed Habitats (June 2015) 1:1,000,000 vector layer for the main gaps, and secondly, EUSeaMap^71^ for a small number of remaining gaps, the latter largely in coastal areas.

Data layers of man-made structures used here comprised:

1. Oil and gas platforms (point locations and details compiled from the Oil & Gas UK (OGUK) Database of North Sea fixed platforms, October 2012 - a product of the North Sea Decommissioning Baseline Study joint industry project (oilandgasuk.co.uk/product/north-sea-decommissioning-database; http://www.insitenorthsea.org/about/); and the OSPAR Offshore Installations Inventory, 2015 (odims.ospar.org)).
2. Wind farms (polygon boundaries from the OSPAR offshore renewables database (odims.ospar.org) with supplementary information, including number of turbines, from RenewableUK (www.renewableuk.com).
3. Wrecks (point locations with details including wreck type, size, material, depth and date sunk, purchased from The Wreck Site - wrecksite.eu).

Summary maps of the natural hard substrate and man-made structure data, processed for use in the particle tracking simulations, are given in Fig. 4. The data layers can be can be obtained from the Cefas Data Hub^72–74^.

### Sedentary communities on offshore structures: selection of species to model

Various studies have investigated the colonisation, establishment and succession of benthic epifaunal communities on artificial substrates^75–78^. Whilst varying species richness has been observed on different substrates ranging from 23 taxa^79^ to 94 taxa^77^ there is a level of consistency within the dominant taxa between studies and their sites of interest. *Mytilus edulis*, *Metridium senile (var. dianthus)*, *Alcyonium digitatum*, *Tubularia spp*. and Porifera spp. are the most notable taxa that are observed on the majority of investigated offshore artificial substrates in north-east Europe.

The taxa selected for simulation in the particle tracking model comprise a selection of those above, augmented with taxa considered of particular ecological importance, such as *Lophelia pertusa* (rare and bioengineering) and *Crepidula fornicata* (non-native and highly fecund). *Echinus esculentus* was included as a representative taxon of natural hard substrate communities. Short summaries of the main life-history characteristics of these species are given in the supplementary material. Detailed information on egg and larval lifecycle stages of the selected species was in some cases difficult to source. Where this was the case, an expert opinion based on information from similar species and species from similar genera was used during the modelling phase of this study. Key factors thought to influence the distribution of the egg and larval phases of the selected species included spawning time, egg/larval duration, vertical migration, size, growth rates and larval stages^31,80^.

### Sectors and connectivity

The offshore structures and natural substrates act both as the spawning and settling sites for the different sedentary species. These species all have a pelagic larvae phase during which they are transported by ocean currents. The resulting connectivity between the different structures depends on this transport and biological behaviour of the species, which was simulated by means of the Lagrangian particle tracking model GITM.

GITM requires a spawning file containing the initial position (latitude, longitude and depth) and time at which each particle is released. Given the number and variety of the available structures, ranging from individual points (oil platforms, wrecks), to polygons (wind farms, natural substrate), we decided to group these structures in sectors of a certain size in order to keep the Lagrangian calculations and posterior analysis at affordable levels.

The “sector approach” consisted of using a regular mesh of fixed size covering the computational domain. Only cells that are occupied by one or several structures were used as release and settling sites. The occupied cells will be called sectors from now on. Connectivity between sectors is assumed to be indicative of connectivity between structures and substrate within them. This sector approach resulted in a significant reduction in the number of release (settling) sites. For this study, we used a multiple of the model grid size to construct the sectors (three times coarser than the original model grid). This choice was a good compromise between the required degree of resolution and the available computational resources. This approach allows for simple, schematic and generalised simulations that allow for maximum flexibility in post processing, such as different scaling for different types of structures.

The wreck data contained information on objects ranging in size from buoys and canoes to ocean liners and oil tankers, including, among others, build type, sinking date, position, tonnage and construction material. To obtain a level of consistency within this and with the oil structures, the wreck data were treated based on tonnage and construction material, as follows. Gaps in the information for tonnage and construction material were filled by using averages of the available data per build type. Objects for which no tonnage could be made available in this way were discarded. Objects made of material based on wood were assumed to have a rapid decay rate above the sea bed and were discarded. In the absence of detailed information on decay, a first order (linear) approximation was assumed for decay due to rot and burial, with a constant decay rate per object, leading to full removal after 150 years: a study in warmer waters, where decay rates may be higher, suggested iron thinning rates in the order of 0.2 mm yr^−1^, and full removal of wrecks within a century^81^. The remaining wreck tonnage was summed per sector. To obtain a significance level for wreck-related connectivity comparable to the oil structures, and to limit the number of sectors to be considered, a threshold weight of 2500 t of wreck was applied to each sector (a typical subsurface oil structure weighs 3500 t).

Maps of the sectors containing natural hard substrate, existing oil structures, wind farms and wrecks (Fig. 4) show that natural hard substrate is mostly confined to coastal areas, oil structures are positioned mostly along the centre line of the North Sea, wind farms are in shallow water, and wrecks are mostly located in coastal and shallow waters. Figure 4 also shows the combined sectors that were considered as spawning (settling) sites in this study.

Once the sectors are defined and the results of the Lagrangian model are available, it is possible to calculate the connectivity among the different sectors. In general, a connectivity matrix *C* is a *MxN* matrix such that the element *C*_*ij*_, *i* = 1,…, *M*, j = 1,…, *N* represents the number of particles that, being released from sector *i*, end up in sector *j*. In this particular study, the connectivity matrix is a square matrix, since the number of source sectors (release sites) is the same as the number of destination sites (settling sites). In order to calculate the connectivity matrix, it is important to define how the process of settlement of a certain particle occurs in terms of settlement time and distance to the settling structure. With respect to the settlement time, we follow a “settlement trace”, meaning that we include all sectors through which a particle passes when it is ready to settle during its final life-history stage as having potential connectivity with the sector from which the particle was released.

The results were processed through the following steps:

1. counting the number of particles settling in each sector (*N*_*settle*_).
2. counting the number of particles supplied by each sector (*N*_*supply*_).
3. scoring the above two roles of each sector on a linear scale from −1 to 1 as a settling/supply factor:
  $$R=\frac{{N}_{supply}-{N}_{settle}}{{N}_{supply}+{N}_{settle}}$$
4. counting the number of sectors from which a sector received (S_rec_)
5. counting the number of sectors to which a sector contributed (S_sup_)
6. classifying sectors into connectivity categories based on 3–5 above (see Table 2).

**Table 2:** Role classification of sectors.

Main role	Settling/supply factor *R*	Supply type	*S*_*sup*_	Reception type	*S*_*rec*_	Category
Suppliers	*R* > 0.5	multi	>5	—	—	8
		mono	≤5	—	*—*	7
Conductors	−0.5 ≤ *R* ≤ 0.5	multi	>5	multi	>5	6
				mono	≤5	5
		mono	≤5	multi	>5	4
				mono	≤5	3
Receivers	*R* <−0.5	—	*—*	Multi	>5	2
		—	*—*	Mono	≤5	1

The resulting categories are: 1: mono-receivers; 2: multi-receivers; 3: mono-suppliers mono-receivers; 4: mono-suppliers multi-receivers; 5: multi-suppliers mono-receivers; 6: multi-suppliers multi-receivers; 7: mono-suppliers; 8: multi-suppliers.

In step 6, we assumed a threshold of 5 as a small number (more than 1, less than 10) to separate mono- and multi-suppliers and receivers.

These connectivity categories can be interpreted in terms of their contribution to the functioning of the network (see Discussion). This procedure was applied to: individual species for each year; individual species aggregated over all 10 years; and all species aggregated across all years. The outcome of each of these steps for one of the species for a single year is presented in the results to illustrate the procedure, with all figures provided in the supplementary material.

### 30-year hindcast and particle tracking experiments

The hydrodynamic model was run from 1 January 1975 to 31 December 1979 from initial conditions consisting of a cold start for tides, and 3D temperature and salinity fields derived from ECMWF-ORAS4. Subsequently for tidal validation, it was run for 6 months in 1980, storing hourly fields, which were subjected to tidal harmonic analysis, resolving a residual, 5 diurnal, 11 semi-diurnal, and 5 shallow-water constituents for elevations and depth-averaged velocity components in the longitudinal and latitudinal directions. A comparison with observed constituents is provided in the supplementary material. After that, the model was run from 1980 until 2010, storing restart files to allow repeat runs for individual years, to store hourly hydrodynamics for the particle tracking simulations. Only about a year of hourly hydrodynamics could be stored, so the subsequent model experiments with particle tracking were carried out year by year for 2001 to 2010; a ten-year period was used to account for inter-annual variations.

For each model experiment, 100 particles were released at the bottom, at the centres of each of the 15 × 15 km grid cell sectors identified as containing structures or hard substrate (67200 particles per model run in total). These numbers were based on a series of preliminary sensitivity runs for sponges with total numbers of particles of 10000, 20000 and 50000 which indicated that for more than 20000 particles the main pattern of connectivity did not change, and only very weak additional connections were established. Bottom releases were used as some of the substrate types considered do not have substantial vertical representation, others have larger representations near the bed (e.g. scour protection), and some of the species considered are not found near the surface even on surface-piercing structures. Release times were randomly generated per particle to occupy bell-shaped curves with a width of one standard deviation straddling the known peak spawning time of the species under consideration (Table 1). Aggregated over 10 years, the population of the bell-shaped curve for the longest standard deviations (1000 particles per sector over 2*90 days) was comparable to that of the sensitivity experiment carried out for the shorter-duration sponges (100 particles per sector over 2*15 days), which showed convergence for more than 20000 particles per annual run. We aimed at maintaining a clear separation between calendar years. Hence, particles with a calculated spawning date before January 1^st^ were released on January 1^st^, this delayed the release of 50% of particles representing Dead man’s fingers larvae to the peak of spawning. Also, runs were terminated on December 31^st^, ignoring the last parts of tracks of a few late-spawned particles representing Plumrose Anemone and Blue Mussels larvae. Particles leaving the domain were taken out of the computation. The life history of particles for each species typically contained an egg stage of fixed duration and a few larval stages with fixed daily growth rates and specific vertical migration behaviours. Growth rates were set as constants and selected to simulate the maximum known larval durations, in order to record the maximum number of possible connections for each particle. The detailed settings of the life-history characteristics of all the species in the model are given in Table 1.

## Data Availability

Connectivity matrices will be made available on the Cefas Data Hub: www.cefas.co.uk/cefas-data-hub.
